# Decision aids that support decisions about prenatal testing for Down syndrome: an environmental scan

**DOI:** 10.1186/s12911-015-0199-6

**Published:** 2015-09-24

**Authors:** Maria Esther Leiva Portocarrero, Mirjam M Garvelink, Maria Margarita Becerra Perez, Anik Giguère, Hubert Robitaille, Brenda J. Wilson, François Rousseau, France Légaré

**Affiliations:** Research Axis of Population Health and Practice-Changing Research, CHU de Québec Research Centre, Saint-François-d’Assise Hospital, 10, rue de l’Espinay, Quebec, QC G1L 3L5 Canada; Department of Epidemiology and Community Medicine, University of Ottawa, 451 Smyth Road, Ottawa, ON K1H 8M5 Canada; Centre d’excellence sur le vieillissement de Québec, CHU de Québec Research Centre, Saint-François-d’Assise Hospital, 10, rue de l’Espinay, Quebec, QC G1L 3L5 Canada; Department of Family Medicine and Emergency Medicine, Pavillon Ferdinand-Vandry, Université Laval, 1050, avenue de la Médecine, Quebec, QC G1V 0A6 Canada; Department of Molecular biology, Medical Biochemistry and Pathology, Faculty of Medicine, Pavillon Ferdinand-Vandry, Université Laval, 1050, avenue de la Médecine, Quebec, QC G1V 0A6 Canada; MSSS/FRQS/CHUQ Research Chair in Health Technology Assessment and Evidence-based Laboratory Medicine, Saint-François-d’Assise Hospital, 10, rue de l’Espinay, Quebec, QC G1L 3L5 Canada

**Keywords:** Prenatal testing, Trisomy 21, Down syndrome, Decision aid, Shared decision making

## Abstract

**Background:**

Prenatal screening tests for Down syndrome (DS) are routine in many developed countries and new tests are rapidly becoming available. Decisions about prenatal screening are increasingly complex with each successive test, and pregnant women need information about risks and benefits as well as clarity about their values. Decision aids (DAs) can help healthcare providers support women in this decision. Using an environmental scan, we aimed to identify publicly available DAs focusing on prenatal screening/diagnosis for Down syndrome that provide effective support for decision making.

**Methods:**

Data sources searched were the Decision Aids Library Inventory (DALI) of the Ottawa Patient Decision Aids Research Group at the Ottawa Health Research Institute; Google searches on the internet; professional organizations, academic institutions and other experts in the field; and references in existing systematic reviews on DAs. Eligible DAs targeted pregnant women, focused on prenatal screening and/or diagnosis, applied to tests for fetal abnormalities or aneuploidies, and were in French, English, Spanish or Portuguese. Pairs of reviewers independently identified eligible DAs and extracted characteristics including the presence of practical decision support tools and features to aid comprehension. They then performed quality assessment using the 16 minimum standards established by the International Patient Decision Aids Standards (IPDASi v4.0).

**Results:**

Of 543 potentially eligible DAs (512 in DALI, 27 from experts, and four on the internet), 23 were eligible and 20 were available for data extraction. DAs were developed from 1996 to 2013 in six countries (UK, USA, Canada, Australia, Sweden, and France). Five DAs were for prenatal screening, three for prenatal diagnosis and 12 for both). Eight contained values clarification methods (personal worksheets). The 20 DAs scored a median of 10/16 (range 6–15) on the 16 IPDAS minimum standards.

**Discussion:**

None of the 20 included DAs met all 16 IPDAS minimum standards, and few included practical decision support tools or aids to comprehension.

**Conclusions:**

Our results indicate there is a need for DAs that effectively support decision making regarding prenatal testing for Down syndrome, especially in light of the recently available non-invasive prenatal screening tests.

**Electronic supplementary material:**

The online version of this article (doi:10.1186/s12911-015-0199-6) contains supplementary material, which is available to authorized users.

## Background

Every year, 447 500 women become pregnant in Canada [1] and are offered prenatal screening to identify serious fetal abnormalities as part of the routine pregnancy care program [2]. The most common fetal anomaly is Down syndrome (DS) which is caused by a trisomy of chromosome 21 (T21) and is characterized by physical problems (such as head and face anomalies, congenital heart defects, gastrointestinal malformation, orthopedic abnormalities, thyroid dysfunction, diabetes mellitus and hearing loss); and behavioral and cognitive problems (including cognitive impairment, attention-deficit/hyperactivity disorder or ADHD), depression, expressive language deficiency, aggressive behavior, and autism [3]. However studies also indicate that individuals with DS can have a fulfilling life and enjoy meaningful relationships and that a significant proportion of parents will choose to pursue a pregnancy with DS [4, 5].

Although all pregnant women are at risk of carrying a fetus with T21, the risk increases with maternal age or with a family history of DS [6, 7]. Prenatal testing is intended to inform women of the risk or presence of certain genetic conditions. Screening is offered to all pregnant women to assess their risk of carrying a fetus with DS, while diagnosis of DS is only offered to women with a positive screening result (indicating that they have a high risk of carrying a child with DS)) [6]. For several years most developed countries have offered both types of prenatal testing for DS: 1) the combined first-trimester screen (non-invasive), and 2) prenatal diagnosis using amniocentesis or chorionic villus sampling (CVS) (invasive). The combined first-trimester screen consists of an ultra-sonographic measurement of nuchal translucency and maternal serum test which measures the levels of β-human chorionic gonadotropin and pregnancy-associated plasma protein A. This test has an 85–90 % detection rate for aneuploidy and a false-positive rate of 5 % [8]. A new non-invasive prenatal screening method (NIPT) requires a simple blood sample from the mother. NIPT uses massive parallel or targeted sequencing of cell-free fetal DNA found in the maternal blood [9–13] and represents an intermediate step between serum screening and invasive diagnostic testing [14]. Although NIPT offers a significant improvement in accuracy, it is not yet offered as a diagnostic test. With greater than 99 % sensitivity and less than a 1 % false-positive rate, however, once its availability is widespread fewer follow-up diagnostic tests will be necessary [8].

Out of 10 000 women who undergo prenatal screening, approximately 415 receive a positive screening result and decide to undergo prenatal diagnosis (amniocentesis or CVS). Of these, 400 will not be carrying a fetus with T21 [7]. However, they may experience moments of anxiety while awaiting their test results (approximately 1–2 weeks) and they face a (small) risk of miscarriage [15]. Indeed, one or two out of the 415 women who undergo amniocentesis will have a miscarriage which could involve a healthy fetus [7, 16], and fetal loss rate for CVS is similar [17–19]. Moreover, DS is not curable, and the test results of a positive diagnosis entail a difficult decision about either terminating the pregnancy or preparing for a high-needs child [20]. Before they reach this stage, therefore, pregnant women need to receive clear and accurate information about the implications of their initial decision about prenatal screening as well as support for values clarification and decision making [21]. The decision to undergo the screening test must be voluntary, well-informed, and congruent with the parents’ values and preferences. Thus, identifying pregnant women’s perceptions about shared decision making is a necessary precursor to promoting their active participation and autonomy in the shared decision making process in the context of both prenatal screening and prenatal diagnosis of DS [22].

Research has shown that pregnant women want to be part of the decision-making process about prenatal tests [23, 24]. Standard information material such as educational leaflets help people to understand their diagnosis and management [25], but in the context of DS this kind of educational material may not be enough to help pregnant women make an informed decision about whether or not to undergo screening [26]. A study on information about DS provided to pregnant women in Canada based on a content analysis of prenatal screening information pamphlets (educational leaflets) concluded that these pamphlets do not present a comprehensive, balanced portrayal of DS and thus are not adequate for supporting shared decision making [27].

Decision aids (DAs) are tools designed to help people participate in decision making about health care options by not only providing information on the options but also by helping them clarify and communicate the personal values they associate with the different options [28]. DAs have been found to stimulate people to take a more active role in decision making, to increase knowledge and, when probabilities are included in DAs, to improve the accuracy of risk perception [29]. A systematic review of 115 studies has demonstrated the effectiveness of DAs in helping people who are facing treatment or screening decisions [29]. Another systematic review showed that DAs can have a positive effect on the decision making process in the prenatal context [25]. More specifically, DAs can significantly decrease decisional conflict [25], defined as a personal uncertainty when making a choice between two or more options that present potential losses or gains, and anticipating potential regret about forgoing the positive aspects of options that were not selected. Decisional conflict is the most commonly used outcome to assess unresolved decisional conflict [30, 31].

Decision aids have been shown to increase knowledge and decrease anxiety with regard to prenatal testing [25]. In addition, provision of detailed information about prenatal testing has been shown to be significantly associated with an increase in patient knowledge and satisfaction [32]. However, not enough studies have evaluated DAs developed specifically for prenatal testing. In fact, according to a systematic review published in 2014 [32], only one study evaluated the effectiveness of DAs for supporting women’s decision making about prenatal testing. A review of decision support technologies for amniocentesis has reported that there is a need for improvement in high-quality publicly available decision support tools [33]. In addition, few new DAs are made publicly available even after studies have proven their effectiveness [34].

Using an environmental scan, we aimed to identify publicly available DAs focusing on prenatal screening/diagnosis for Down syndrome. Then, using IPDAS minimum standards as well as data on practical support and comprehensibility, we aimed to analyze the extent to which these DAs could support decision making in the context of prenatal testing for DS.”

## Methods

The 16 Minimum Standards for Certifying Patient Decision Aids in the refined International Patient Decision Aids Standards (IPDASi v4.0) represent essential elements of information that must be present in any DA [35]. However, they do not indicate the presence of practical decision support tools, such as methods for evaluating women’s understanding of the information about the test and of options and outcomes, or values clarification methods, both of which are additional important elements of effective DAs [28, 29]. Values clarification methods are sections within DAs intended to assist patients in elucidating their values and preferences about the options so they can fully integrate the information and finally make an informed choice that is in accordance with their values and preferences [36]. These methods consist of worksheets that lead patients through the three steps (choosing, prizing and acting) of the values clarification process, [37] and present the options in a balanced way to help patients weigh their relative benefits and limitations [36, 38]. In addition, the minimum standards do not address comprehensibility of the information, such as visual representations (graphs, tables, drawings, pictures, organizational charts, algorithms) or educational components (glossary, definitions, diagrams, abbreviations or explanations, tutorials, links, flowcharts). Based on the expanded IPDAS checklist for users [39], we therefore extracted data concerning these additional components.

An environmental scan (ES) was therefore performed in order to identify all publicly available DAs that focus on prenatal screening/diagnosis for DS. The IPDAS minimum standards and data on their practical and comprehension features were used to explore the extent to which these DAs could support decision making.

Environmental scans were developed as tools for retrieving and organizing data from a wide variety of fields in order to identify contexts and shifts in planning for the future [40]. They can include internal (e.g. memos, notes from meetings with stakeholders, etc.) as well as external sources (e.g. newly available technologies) [40]. An environmental scan was appropriate because we sought to identify as many decision aids in this area as possible, irrespective of whether or not they had been the subject of published evaluations. Given that most of the DAs we identified had not been evaluated, they would have been missed in a systematic review [40]. DAs developed by our team were considered as an internal source in addition to DAs developed by other institutions, thus increasing the number of DAs available for analysis.

The scan followed the PRISMA flow diagram for reporting standards in systematic reviews and meta-analysis [41]. Ethics approval was obtained from the Research Ethics Boards of the Centre de Santé et Services Sociaux de la Vieille-Capitale (#2013-2014-29) in Quebec, and the CHU de Quebec (#B14-02-1929) as a part of the PEGASUS Project.

### Data sources and search strategies

Four main data sources were searched: i) The Decision Aids Library Inventory (DALI) of the Ottawa Patient Decision Aids Research Group at the Ottawa Research Institute from September 16, 2013 to April 20, 2014. The DALI contains an up-to-date overview of freely available DAs on several health topics that meet a minimal set of certification and qualification criteria based on the IPDASi v4.0 (International Patient Decision Aid Standards) [35]; ii) from February to May 2014, we used our research network to contact professional organizations, academic institutions, and experts in the shared decision making (SDM) field (email, Facebook group publications, the shared@each SDM network). Experts were asked if they had produced DAs on prenatal testing for DS or if they knew other researchers who may have done so; iii) from May 26 to June 4 2014, with the help of Google, electronic databases such as YouTube were searched using the following search strategies designed by an information specialist and expert librarian: (decision aid) and (prenatal screening) and (trisomy 21); (support tool) and (prenatal screening) and (trisomy 21); (prenatal screening program) and (trisomy 21); (decision aid) and (prenatal screening) and (Down syndrome); (support tool) and (prenatal screening OR prenatal screening program) and (trisomy 21 OR Down syndrome). The first 150 sites found with each search strategy were analyzed to see if they met the eligibility criteria; and finally iv) the references in existing systematic reviews on DAs were reviewed [42]. If DAs were not available, a copy was requested from their developers by email.

### Data selection

DAs were included that met the following inclusion criteria: a) targeted pregnant women, b) focused on prenatal testing (screening and/or diagnosis), c) applied to tests for fetal abnormalities or aneuploidies, d) were in French, English, Spanish or Portuguese.

The age of the DA was not an inclusion criterion because we wanted to capture the greatest number of DAs possible, and hypothesized that there were few DAs to support women in prenatal screening for DS [25]. Furthermore, DA updating is one of the 16 minimum criteria IPDASi (v4.0) and thus any selection bias was avoided.

All DAs were screened independently by two reviewers (MMBP, MELP) to determine if they met these four inclusion criteria. Eligibility was determined by consensus and any discrepancies were discussed with the project coordinator (HR) and the research team. Kappa coefficient was computed to measure final agreement among reviewers on DA selection.

### Data extraction

Using a data extraction grid developed by our team (available from authors), two reviewers (MMBP, MELP) independently extracted the following characteristics of the eligible DAs: title; country of origin, language; date of creation/publication; name of developer, author or editors; website where it could be accessed; whether it was freely available; targeted public; types and nature of the tests included; and genetic abnormalities detected by the included tests. Features of each DA relevant to practical decision support and comprehensibility were also extracted. Second, DAs were independently assessed by two reviewers using a grid created by our team (available from authors) based on the IPDAS minimum standards. Six “qualifying” criteria determine whether the intervention can be considered a DA and 10 “certification” criteria determine risk of harmful biases [35]. Each item scored either 0 (criterion not met) or 1 (criterion met). A total score was obtained by adding up the scores on all items, and ranged from 0 to 16.

Results of the data extraction were determined by consensus. Any discrepancies were resolved through discussion among reviewers and with the project coordinator (HR) and the research team. Kappa coefficient was computed to measure final agreement among reviewers.

### Data synthesis and analysis

A narrative synthesis of the data was performed, with assessments of frequency counts of all characteristics identified including the presence of practical decision support tools and comprehension aids. Simple descriptive statistics were used to describe eligible DAs and their quality according to the IPDASI V4.0 minimum standards.

## Results

### DA selection

**Fig. 1:**
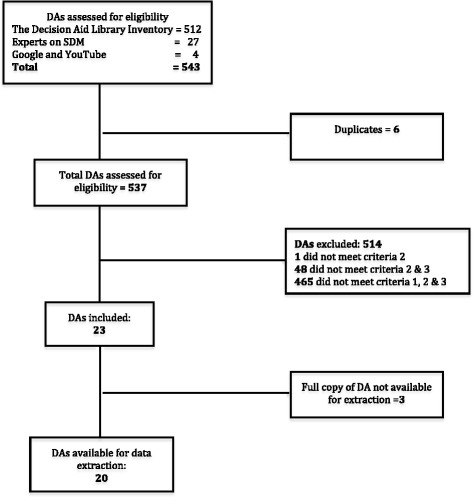
Flow diagram of decision aid selection. Criteria 1: DA targets pregnant women; Criteria 2: DA focuses on prenatal testing (screening and/or diagnosis); Criteria 3: DA applies to tests for fetal abnormalities or aneuploidies; Criteria 4: DA in French, English, Spanish or Portuguese

Figure 1 illustrates the flow of the DAs selection process. Out of a total of 537 unique potentially eligible DAs, 23 met the eligibility criteria, 20 of which were available for data extraction. Kappa coefficient was 1.0. Table 1 shows the 20 eligible DAs listed in alphabetical order.

**Table 1:** DAs that met eligibility criteria

	Title	Developer	Year of publication	Source
a	A Decision Aid: Testing in Pregnancy for foetal Abnormalities	Murdoch Children’s Research Institute, Australia	2004	DALI - Ottawa Research Institute
b	Amniocentesis	Option Grid Collaborative, Dartmouth, USA	2012	DALI - Ottawa Research Institute
c	Amniodex: Amniocentesis Decision Explorer	Decision Laboratory, University of Cardiff, Wales, UK.	2008	DALI - Ottawa Research Institute
d	Antenatal Down Syndrome Screening (VIMEO)	Department of Obstetrics and Gynecology, Stockholm, Sweden.	2011	DALI - Ottawa Research Institute
e	Choices about first trimester ultrasound scans: A decision aid for pregnant women	Queensland Centre for Mothers & Babies - University of Queensland, Australia	2010	Google
f	Do I want a screening test for Down syndrome	The Newcastle upon Tyne Hospitals NHS, UK	2013	SDM Organisations
g	Down Syndrome Screening	Option Grid Collaborative, Dartmouth, USA	2013	DALI - Ottawa Research Institute
h	Information destinée aux femmes enceintes sur la possibilité de recourir’a leur demande au dépistage prénatal de la trisomie 21	Collège national des gynécologues et obstétriciens français, France	2012	Google
i	Is my baby alright	Midwives Information and Resource Service, UK	1997	DALI - Ottawa Research Institute
j	Making choices: Prenatal Testing	Ottawa Patient Decision Aid Research Group, Canada	1999	DALI - Ottawa Research Institute
k	Pour les femmes et leurs familles. Un guide pour mieux comprendre les tests de dépistage prénatal	Mount Sinai Hospital, Toronto, Canada	2007	SDM Experts
l	Pregnancy - should I have screening tests for birth defects?	Healthwise, USA	2013	DALI - Ottawa Research Institute
m	Pregnancy - should I have CVS?	Healthwise, USA	2012	DALI - Ottawa Research Institute
n	Pregnancy, should I have amniocentesis?	Healthwise, USA	2012	DALI - Ottawa Research Institute
o	Pregnancy - should I have an early fetal ultrasound?	Healthwise, USA	2012	DALI - Ottawa Research Institute
p	Prenatal diagnosis for Down Syndrome	University of Leeds, UK	2004	DALI - Ottawa Research Institute
q	Prenatal Screening - Is it Right for You?	Dartmouth - Hitchcock, USA	2013	SDM Experts
r	Prenatal Screening Video	Michigan State University, USA	2007	YouTube
s	The California Prenatal Screening Program	California Department of Public Health - Genetic Disease Screening Program - Prenatal Screening Program, USA	2013	Google
t	Ultrasounds scans - What you need to know	Midwives Information and Resource Service, UK	2008	DALI - Ottawa Research Institute

### Characteristics of DAs

Additional file 1: Table S1 summarizes characteristics of the DAs retained.

#### Date, place of origin, cost

The DAs identified were developed between 1996 and 2013 in the United Kingdom (7; 35 %), United States of America (7; 35 %), Canada (2; 10 %), Australia (2; 10 %), Sweden (1; 5 %) and France (1; 5 %). Most of the DAs were in English (17; 85 %). Two DAs (10 %) were not freely available but could be bought online for £3.60 (Additional file 1: Table S1).

#### Purpose & target of DAs

Five DAs (25 %) were for prenatal screening only, three (15 %) for prenatal diagnosis and 12 (60 %) for both. The prenatal screening tests included in the DAs (not mutually exclusive) were: ultrasound scan (14; 70 %), maternal serum (12; 60 %), nuchal translucency (11; 55 %) and Non-Invasive Prenatal Testing (NIPT) (1; 5 %). All 20 DAs targeted pregnant women, five DAs also targeted their partners, and two also targeted healthcare professionals (Additional file 1: Table S1).

#### Practical tools for decision support

One DA provided a content summary (5 %); eight (40 %) included values clarification methods; and five (25 %) provided a method for evaluating women’s understanding of the information provided (about the test, the options and/or of the outcomes) (Additional file 1: Table S1). Overall, a median of 8/20 provided practical decision support methods (personal worksheets).

#### Content comprehensibility

All DAs provided some kind of visual representation. DAs used graphs (5; 25 %), tables (14; 70 %), drawings (7; 35 %), pictures (11; 55 %), organizational charts (1; 5 %) or algorithms that clearly showed the steps of the decision process (1; 5 %). All DAs provided some kind of educational component, including important definitions (17; 85 %), a glossary (1; 5 %), a diagram (1; 5 %), explanations and/or abbreviations (8; 40 %), links to more information (14; 70 %) or a flowchart of the decision making process (1; 5 %).

### IPDAS minimum standards

With regard to our IPDAS “qualifying” criteria (Table 2), all 20 DAs (100 %) explicitly stated the index decision and 17 (85 %) described the health condition or problem for which the index decision was required. Sixteen (80 %) described the options available for the index decision, among which eight (40 %) described both the positive and the negative features of each option (to do or not to do the test). With regard to “certification” criteria, all 20 DAs (100 %) described what the prenatal test was designed to measure. Half of the DAs (50 %) provided information about an update policy, and eight (40 %) (not mutually exclusive) showed the negative and positive features of options in equal detail (using similar fonts, sequence, and representation of statistical information).

**Table 2:** Quality assessment of DAs (*n* = 20) according to the IPDASi v4 criteria

Dimension	Item		a	b	c	d	e	f	g	h	i	j	k	l	m	n	o	p	q	r	s	t	Total number of DAs fulfilling the criterion (out of 20)
Qualifying criteria																							
Information	1	DA describes health condition or problem for which index decision is required	X		X	X	X			X	X	X	X	X	X	X	X	X	X	X	X	X	17
	2	DA explicitly states the decision that needs to be considered (index decision)	X	X	X	X	X	X	X	X	X	X	X	X	X	X	X	X	X	X	X	X	20
	3	DA describes the options available for the index decision	X	X	X	X	X	X	X		X	X	X	X	X	X	X	X				X	16
	4	DA describes the positive features (benefits/advantages) of each option	X					X				X		X	X	X	X	X					8
	5	DA describes the negative features (harms, side effects, or disadvantages) of each option	X	X				X	X			X		X	X	X	X	X					10
Values	6	DA describes what it is like to experience the consequences of the options (physical, psychological, social)	X	X	X	X	X				X		X	X	X	X	X	X	X	X			14
Certification criteria																							
Information	7	DA shows the negative and positive features of options in equal detail (using similar fonts, sequence, and representation of statistical information)	X					X				X		X	X	X	X	X					8
Evidence	8	DA (or associated documentation) provides citations to the evidence selected		X	X				X			X		X	X	X	X	X					9
	9	DA (or associated documentation) provides a production or a publication date	X	X	X	X		X	X	X	X	X	X						X	X	X	X	14
	10	DA (or associated documentation) provides information about the update policy		X			X	X	X		X			X	X	X	X					X	10
	11	DA provides information about the levels of uncertainty around event or outcome probabilities	X	X	X	X		X	X	X	X	X	X	X	X	X	X	X	X	X	X	X	19
Disclosure	12	DA (or associated documentation) provides information about the funding source used for development	X	X	X				X		X			X	X	X	X					X	10
Test	13	DA describes what the test is designed to measure	X	X	X	X	X	X	X	X	X	X	X	X	X	X	X	X	X	X	X	X	20
	14	DA describes the next steps typically taken if the test detects the condition or problem	X	X	X	X		X	X	X	X	X	X	X	X	X	X	X	X	X	X	X	19
	15	DA describes the next steps if the condition or problem is not detected	X			X											X	X					4
	16	DA has information about the consequences of detecting the condition or disease that would never have occurred if screening had not been done (lead time bias)												X			X						2
Total quality score (out of 16)^a^			13	11	10	9	6	10	10	6	10	11	8	14	13	13	15	12	7	7	6	9	

^a^minumum-maximum scores ranges from 0 to 16

None of the 20 identified DAs met all minimum standards. The median score on the 16 Minimum Standards for Certifying Patient Decision Aids in the refined International Patient Decision Aids Standard (IPDASi v4.0) for the 20 DAs was 10 out of 16 points (range 6–15). Kappa coefficient was 1.0.

## Discussion

We conducted an environmental scan to identify existing DAs focusing on prenatal testing for DS that could support decision making about taking or not taking the test. We identified 23 DAs of which 20 were available for data extraction. They were produced between 1996 and 2013, in six countries and in three languages. The principal disorder targeted by all the identified DAs was DS. Seventeen DAs were for prenatal screening, either solely or in combination with prenatal diagnosis, and three were for prenatal diagnosis only. Very few eligible DAs targeted a spouse or partner or supported a conversation with a provider. Few contained practical decision support tools and none fulfilled all the IPDAS minimum standards criteria. The median for the 20 DAs appraised was 10 out of 16 points. Our results lead us to make four main observations.

First, the aim of DAs in the context of prenatal testing is to help pregnant women make values-sensitive decisions involving not only their own health, but also the health of their fetus. DAs should provide unbiased nondirective scientific information on the risk and benefits of all options, and assist pregnant women in clarifying their personal values concerning outcomes and adverse effects [25, 39, 43]. At the moment DAs are not achieving this purpose. Although DALI considers all the tools in its library as DAs, based on our scoring using the IPDAS there is large variation in quality across the included DAs whether they are listed in DALI or not. In addition, although the available DAs contained some of the information necessary for SDM, our analysis showed that few contained the tools necessary for putting it into practice. The majority mentioned that pregnant women’s personal values were important for decision making, but less than half helped the women express their uncertainties about the available options by providing a values clarification instrument, or provided a tool for assessing their understanding. In addition, only one DA has been evaluated for its effectiveness in fostering SDM in practice [44].

Second, although the DAs in this study were all available, some were difficult to obtain. Moreover, we have no data with regard to the frequency with which they are actually used in practice. Indeed, studies show that numerous DAs have been developed and evaluated but not made publicly available afterwards. Although these DAs were not the focus of this study, this failure to reach the public should receive adequate attention in future studies [34]. This highlights the need to pay close attention to implementation strategies in the development of any new DA about prenatal testing [45].

Third, all the DAs we found were produced after 1996, which is in keeping with the period in which prenatal tests became available [46]. However, of the 20 DAs included in our study, 11 (55 %) were produced after 2011. As the 16 IPDASi(v4.0) minimum criteria [35] were only established in 2012, these minimum criteria may not have been used as a development guide, although the 47 IPDASi(v3.0) criteria [47] established in 2008 included the 16 minimum criteria. This leads us to believe that DA authors post-2008 were either not aware of IPDAS at all, as this is a young field, or did not use the criteria effectively.

Fourth, interestingly, many DAs were developed in the same country, language and period. In the United States, for example, six prenatal testing DAs were published between 2012 and 2013 and in the United Kingdom three were published in the same period. On the one hand their development coincides with the availability of prenatal testing in their respective healthcare systems, but their number also illustrates a lack of internal collaboration on development of important patient resources. Future studies should emphasize collaborative development of publicly available patient information and decision support, thereby focusing scarce resources on supporting pregnant women in making these difficult decisions instead of on choosing which of several available DAs to use.

These results should be interpreted with caution due to some limitations [40]. The search strategy was limited to four main sources: the Decision Aids Library Inventory, SDM experts, Google and the references in existing systematic reviews on DAs. As the focus was only on DAs available to the general public, some may have been missed. For example, developmental or evaluation studies that publish quality assessment data on DAs that are not publicly available were not searched. Data was restricted to publicly available DAs since these are what the client will see and use, and are thus the most relevant to supporting informed decision making in practice today. Hence, although the four data sources did not permit the capture of tools not explicitly labelled “decision aids”, they did allow capture of the majority of existing too.

## Conclusion

According to one systematic review, the number of pregnant women who undergo prenatal screening is slightly higher among women informed with DAs, so they do have some impact on informed choice in pregnancy care [25]. Nevertheless, according to the results of our analysis of DAs, at this point in time none of them offer proper support to pregnant women in decision making about prenatal screening/diagnosis for DS. The quality of identified DAs did not meet the IPDAS minimum standards, and few contained the tools needed to support informed decision making in practice. We therefore recommend that DAs for prenatal testing that consider the 16 minimal standards recommended by the IPDAS, ensure comprehensibility and contain practical decision support tools. In addition, attention should be paid to current developments in prenatal testing techniques, such as non-invasive prenatal testing (NIPT), which was addressed in one DA only.

We consider that our study will contribute to the development of higher-quality decision support tools in the future, as well as to the implementation of shared decision making in the context of the sensitive decisions about prenatal screening for DS.

Based on the outcomes of this study we are currently developing a DA to support the decision about prenatal testing for DS. The next phase in its development will be its evaluation among women facing this decision.

## Additional file

Additional file 1: Table S1.
**Data extraction grid consensus.** (PDF 35 kb)

## Abbreviations

ADHD: Attention-deficit/hyperactivity disorder
DA: Decision aid
DALI: Decision aids library inventory
DS: Down syndrome
IPDAS: International patient decision aids standards
NIPT: Non-invasive prenatal testing
SDM: Shared decision making
