# Supplementary material for: Decision aids that support decisions about prenatal testing for Down syndrome: an environmental scan
Source: BMC Med Inform Decis Mak. 2015 Sep 24;15:76. doi: 10.1186/s12911-015-0199-6 (PMC4583147; doi:10.1186/s12911-015-0199-6)
Supplement: Additional file 1: Table S1. — Data extraction grid consensus. (PDF 35 kb) [file 12911_2015_199_MOESM1_ESM.pdf]

SUPPLEMENTARY TABLE 1 - DECISION AIDS IN THE CONTEXT OF PRENATAL SCREENING OF DOWN SYNDROME - Data Extraction Grid CONSENSUS

Specific items		Title of decision aid	a	b	c	d	e	f	g	h	i	j	k	l	m	n	o	p	q	r	s	t	Total
Main characteristics		Country	Australia	UK	UK	Sweden	Australia	UK	UK	France	UK	Canada	Canada	USA	USA	USA	USA	UK	USA	USA	USA	UK	UK = 7, USA = 7, Canada = 2, Australia = 1, Sweden = 1, France = 1
		Language	English	English	English	English subt.	English	English	English	French	English	English	English, French	English	English	English	English	English	English	English	English, Spanish	English	English = 16, French = 1, English/French = 1, English/Spanish =1
		First publication	nd	nd	2000	nd	nd	nd	nd	juin-12	janv-97	1999	août-07	nd	nd	nd	nd	2004	2012	2007	02 - 2013	janv-96	Jan 1996 - April 2012
		Update	2004	2012-12-05	2008	nd	March 2010	May 2013	2013-01-13		sept-08			2013-05-28	2012-04-04	2012-04-04	2012-06-18	nd				sept-08	2004 - 2013
		Format	web,pap,pdf	web,pap,pdf	web,paper	video (web)	pap. Online	web,pap,pdf	web,pap,pdf	paper, printable	pap,pdf,leaflet	paper	web,pap,pdf	web,paper	web,paper	web,paper	web,paper	web,paper	pap,pdf (web), video	video (web)	paper, printable	pap,pdf,leaflet	
		No. of pages	30	1	54		17	2	1	5	12	38+2	12	10 + 2	9 + 2	10 + 2	9 + 2	5	31		19	12	1-54
		Developer	M.C.R.I.	Option grid	Cardiff Univ.	Karolinska univ	Centre for Mothers & Babies - University of Queensland	NHS Foundation Trust	Option grid	Collège National des Gynécologues et Obstétriciens Français	MIDIRS	OPDARG	Mount Sinai Hospital	Healthwise	Healthwise	Healthwise	Healthwise	Univ Leeds	Dartmouth-Hitchcock Medical	Department of Pediatrics and Human Development	California Department of Public Health, Genetic Disease Screening Program	MIDIRS	
		Website	mcri.edu.au	optiongrid.org	amniodex.com	vimeo.com/ 34600615	http://www.havingababy.org.au/media/pdf/firsttrimesterultrasound.pdf	http://personcentredcare.health.org.uk/resources/brief-decision-aids	optiongrid.org	http://www.cngof.asso.fr//D_TELE/120614_INFORMATION_TRISOMIE_21.pdf	infochoice.org	decisionaid @ohri.ca	http://www.nygh.on.ca/data/2/rec_docs/301_Prenatal_Screening_PatientInfo_French.pdf	healthwise.net	healthwise.net	healthwise.net	healthwise.net	http://www.google.ca/url?sa=t&rct=j&q=&esrc=s&source=web&cd=1&ved=0	http://patients.dartmouth-hitchcock.org/obstetrics/prenatal_testing_genetic_counseling.html	http://www.phd.msu.edu/divisions/humangenetics/prenatal-screening-video.html	http://www.cdph.ca.gov/programs/pns/pages/default.aspx	infochoice.org	20
		Free	yes	yes	yes	yes	yes	yes	yes	yes	no	yes	yes	yes	yes	yes	yes	yes	yes	yes	yes	no	18
		Cost									£ 3,60											£ 3,60	
Description	Test classification	Prenatal screening	yes		yes	yes	yes	yes	yes	yes	yes	yes	yes	yes	yes		yes		yes	yes	yes	yes	17
		Prenatal diagnosis	yes	yes	yes	yes				yes	yes	yes	yes		yes	yes	yes	yes	yes		yes	yes	15
		Others																					
	Type of test	Maternal serum	yes		yes	yes			yes	yes	yes	yes	yes	yes					yes	yes	yes	yes	13
		Ultrasound scan	yes		yes	yes	yes		yes	yes	yes	yes	yes	yes			yes		yes		yes	yes	14
		Amniocentesis	yes	yes	yes	yes				yes	yes	yes	yes			yes		yes	yes		yes	yes	13
		Chorionic Villus sampling	yes			yes				yes	yes	yes	yes		yes			yes	yes		yes	yes	11
		Nuchal translucency	yes		yes	yes				yes	yes		yes	yes					yes	yes	yes	yes	11
		NIPT																	yes				1
		Others	combined test		PCR,kariotype	CUB combined		Unclear			combined test											CRL	
	Abnormality classification	Physical abnormalities	yes			yes	yes				yes	yes	yes	yes	yes	yes	yes		yes	yes	yes	yes	14
		Chromosomal abnorm.	yes	yes	yes	yes	yes	yes	yes	yes	yes	yes	yes	yes	yes	yes	yes	yes	yes	yes	yes	yes	20
		Others																					
		Unclear																					
	Abnormality testing	Down syndrome T21	yes	yes	yes	yes	yes	yes	yes	yes	yes	yes	yes	yes	yes	yes	yes	yes	yes	yes	yes	yes	20
		Edward syndrome T18	yes	yes	yes							yes	yes	yes					yes	yes	yes		9
		Patau syndrome T13	yes	yes	yes									yes							yes		5
		Sex chrom. abnorm.	yes																				1
		Neural tube defects	yes			yes					yes		yes	yes		yes	yes		yes	yes	yes	yes	11
		Card, dig, ren. Abnorm.	yes			yes	yes				yes			yes								yes	6
		Others												genetic problems	Tay-Sachs, hemophilia	Cystic fibrosis, Huntington's d, sickle cell d.				Open fetal defects, Smith-Lemli-Opitz Syndrome, Cystic	Abdominal wall defects, Smith-Lemli-Opitz Syndrome (SLOS), SCD	genetic problems	
	Targeted public	Pregnant women	yes	yes	yes	yes	yes	yes	yes	yes	yes	yes	yes	yes	yes	yes	yes	yes	yes	yes	yes	yes	20
		Partners		yes	yes	yes			yes				yes										5
		Health prof.		yes					yes														2
		Others																					
Practical usefulness for SDM		Summary										yes											1
		Uncertainty explicit	yes				yes					yes		yes	yes	yes	yes						7
		Personal worksheet	yes				yes					yes		yes	yes	yes	yes		yes				8
		Women's understanding	yes											yes	yes	yes	yes						5

Comprehensibility	Visual Representations	Graph	yes		yes		yes					yes								yes			5
		Table	yes	yes				yes	yes			yes	yes	yes	yes	yes	yes	yes	yes	yes	yes		14
		Drawing	yes		yes							yes	yes						yes	yes	yes		7
		Picture	yes		yes	yes	yes				yes	yes	yes			yes			yes	yes		yes	11
		Organ.chart																yes					1
		Algorithm								yes													1
		None																					
		Others											Prenatal screening calendar							reasoning of other patients			
	Educational components	Glossary																				yes	1
		Definitions	yes		yes	yes	yes			yes	yes	yes	yes	yes	yes	yes	yes	yes	yes	yes	yes	yes	17
		Manual																					
		Diagram			yes																		1
		Abrev.explanations	yes				yes			yes	yes		yes						yes		yes	yes	8
		Tutorials																					
		Links	yes	yes	yes	yes	yes	yes	yes	yes	yes		yes					yes	yes		yes	yes	14
		Flowchart			yes																		1
		None																					
		Others	Summary of tests, table of contents, scale		14 videos, board index	parents' stories (witness)					Sheet for writing questions			women's stories, scales	women's stories, scales	women's stories, scales	women stories, scales					Sheet for writing questions	

M.C.R.I.=Murdock Children's Research Institute
web= internet site
pap= paper
pcr= polymerase chain reaction
cub= combined ultrasound and biochemica
d=disease
card dig ren abn=
